# Social media use and adolescents’ well-being: A note on flourishing

**DOI:** 10.3389/fpsyg.2023.1092109

**Published:** 2023-04-06

**Authors:** Laura Marciano, Kasisomayajula Viswanath

**Affiliations:** Harvard T. H. Chan School of Public Health, Lee Kum Sheung Center for Health and Happiness, Dana-Farber Cancer Institute, Boston, MA, United States; Institute of Public Health, USI Università della Svizzera italiana, Lugano, Switzerland

**Keywords:** social media, adolescence, flourishing, self-determination theory, self-disclosure, inspiration

## Abstract

**Background:**

Several large-scale studies and reviews have reported both negative and positive associations of social media use with well-being, suggesting that the findings are more complex and need more nuanced study. Moreover, there is little or no exploration of how social media use in adolescence influences *flourishing*, a more all-encompassing construct beyond well-being, including six sub-domains (i.e., happiness, meaning and purpose, physical and mental health, character, close social relationships, and financial stability). This paper aims to fill this gap by understanding how adolescents might flourish through social media activities by fulfilling the basic needs pointed out by the Self-Determination Theory, i.e., relatedness, autonomy, and competence.

**Methods:**

The study is drawn on cross-sectional data collected from 1,429 Swiss adolescents (58.8% females, M_age_ = 15.84, SD_age_ = 0.83) as part of the HappyB project in Spring 2022. Self-reported measures included the Harvard Adolescent Flourishing scale, positive and negative online social experiences, self-disclosure on social media, and social media inspiration. Control variables included, among others, self-esteem, ill-being, and personality.

**Results:**

After applying Bonferroni’s correction, results of the hierarchical regression analyses showed that positive social media experiences (β = 0.112, *p* < 0.001) and social media inspirations from others (β = 0.072, *p* < 0.001) and for others (β = 0.060, *p* = 0.003) were positively associated with flourishing. Flourishing was inversely associated with negative social media experiences (β = −0.076, *p* < 0.001). Among covariates, self-esteem (β = 0.350, *p* < 0.001), ill-being (β = −0.252, *p* < 0.001), perceived school environment (β = 0.138, *p* < 0.001), self-reported level of physical activity (β =0.109, *p* < 0.001), and perceived socio-economic status (β = −0.059, *p* = 0.001) were all related to flourishing. In contrast, gender, high school year, age, perceived stress, and personality (extraversion and neuroticism) were not.

**Conclusion:**

Using a well-being framework to investigate social media use in adolescents is needed to go beyond the ill-being perspective. Our results align with the needs pointed out by the Self-Determination Theory. Carrying out social media activities in a way that promotes—rather than diminishes—flourishing should be included as an additional good habit influencing adolescents’ development. We suggest that interventions aiming to foster adolescents’ flourishing should include curricula aiming to promote a good use of social media through positive online social relationships and inspirational contents.

## Introduction

In Switzerland, 99% of 12 to 19-year-olds own a smartphone, with higher prevalence rates in late adolescence (Süss et al., 2020). The most frequent activities include instant messaging and social media use (Süss et al., 2020). Compared to 2018, in 2020, Swiss adolescents spent 40 min more on their smartphones during a typical weekday (achieving a total of more than 3 h/day) and almost 2 h more during a weekend day (for a total of about 5 h/day). This was the highest level since 2010 (Süss et al., 2020), though, likely, partly due to the ongoing COVID-19 pandemic (Fernandes et al., 2020; Montag and Elhai, 2020). Indeed, due to COVID-19, increased time spent on social media was reported, together with a higher prevalence of problematic use of digital technologies, and these increments have been related to adverse health outcomes (Marciano et al., 2022a), including an increase in depressive symptoms, anxiety, and inattention problems (Marciano et al., 2022b). Several large-scale studies showed that adolescents who spend more time on screens also show lower overall well-being (Twenge and Campbell, 2019; Twenge et al., 2021), lower life satisfaction and happiness (Booker et al., 2015; Twenge et al., 2018), as well as higher levels of loneliness (Kelly et al., 2018; Twenge et al., 2021) and depression (Kelly et al., 2018; Twenge and Campbell, 2018). However, four reviews of reviews (Dickson et al., 2018; Odgers and Jensen, 2020; Orben, 2020; Valkenburg et al., 2021) highlighted small associations between digital media use and adolescents’ well-being in both negative and positive directions, thus leading to mixed results and to the conclusion that *there is no uniform effect for all adolescents*. The presence of positive associations with social media use—in addition to the negative ones—also aligns with the digital Goldilocks hypothesis (Przybylski and Weinstein, 2017), according to which a moderate—“just right”—use of digital technologies would not be intrinsically harmful. In other words, when digital media use is too high, that might be at the expense of other offline activities (Camerini et al., 2020). If, on the other side, it is too low, it can deprive young people of gaining important information and connecting with peers. For example, Twenge et al. (2018) reported that, compared with no use at all, adolescents’ levels of happiness were higher when digital media were used for at least a few hours a week. Similarly, digital detox would cut off young people from social connections and support, thus lowering well-being and making people crave even more to spend time online when the detox period is over (Radtke et al., 2022).

The evaluation of well-being in adolescence is particularly crucial, considering that half of the mental health disorders with long-lasting effects start in mid-adolescence (Kessler et al., 2005, 2007; Merikangas et al., 2010). A significant increase in the prevalence of adolescents’ mental health problems has been reported in the past decade in the United States (Monaco, 2021) and Europe (Neufeld SAS, 2022), thus representing a great public health concern. In Switzerland, from 2012 to 2020, people treated for mental health problems increased by approximately 26% in total, with higher growth (+40%) in children and adolescents than in adults (+25%; Federal Statistical Office, 2022). This increase has been related to changes in epigenetic factors, i.e., modifiable factors deriving from the continuous and dynamic interconnection of genetic predispositions with environmental situations, with social media and screen time as one of the factors proposed to have contributed to such a growth (Monaco, 2021). Yet, more research should be conducted to explore *well-*being outcomes. Indeed, well-being is not only the absence of mental illness, but it is related to the presence of happiness, having a purpose and a sense of meaning, and good relationships (VanderWeele et al., 2020). According to the positive psychology and epidemiology perspectives, well-being is “a complex construct that concerns optimal experience and functioning” (Ryan and Deci, 2001). For some authors, well-being cannot just be defined bi-dimensionally on a continuum, but it is multifaceted, like a “garden” or an “orchestra” (Lomas and Vanderweele, 2022). Today, the concept of flourishing (Henderson and Knight, 2012; Huppert, 2013; Willen, 2021) mirrors these multi-facets by measuring a sense of growing and prospering, and it has been pointed out as a new conceptual framework for defining well-being (Huppert, 2013). Studying flourishing is becoming crucial to move from the ill-being framework (i.e., the study of psychopathological symptoms) to the science of promoting a broader notion of human well-being as an important means through which society can thrive (VanderWeele, 2017). In one review of 11 studies focusing on adolescence (Witten et al., 2019), flourishing has been conceptualized in diverse and vague ways. In particular, the presence of different assessment instruments does not fully align with the wide meaning of the construct since they often measure only one component of flourishing, like life satisfaction or meaning. In the present study, we drew on the definition by VanderWeele (2017), who refers to flourishing as “a state in which all aspects of a person’s life are good” (p. 8149), thus capturing a holistic view of what it means to thrive. In particular, flourishing would include six broad domains, i.e., happiness and life satisfaction (hedonic well-being), meaning and purpose (eudaimonic well-being), physical and mental health, character and virtue, close social relationships (social well-being), and financial stability (VanderWeele et al., 2019). With the exception of financial well-being, these domains are usually viewed as ends and almost universally desired. Whereas financial stability is not an end, but it enables to preserve goods that are their own ends. Also, although not exhaustive, four pathways are associated with the domains of flourishing, i.e., family, work, education, and religious community (VanderWeele, 2017). These pathways highlight the positive role of supportive family relations and marriage, employment vs. unemployment, higher education (with possibly greater effects in the United States than European countries), and participation in religious community, respectively, on improving flourishing in the adult population. Yet, how the online environment relates to flourishing in adolescents is still largely unexplored. For example, adolescents might flourish through social media activities by fulfilling the basic needs pointed out by the Self-Determination Theory (SDT Ryan and Deci, 2000; Huppert, 2013), i.e., relatedness, autonomy, and competence. According to the STD, these needs drive human motivation and should be met for optimal development and functioning. Indeed, adolescents need to feel a sense of closeness and connectedness with others to experience emotional security and acceptance; also, they need to develop their sense of autonomy and identity by expressing themselves and making choices freely; finally, adolescents want to feel competent by showing their skills and achieving chosen goals effectively (Hui and Tsang, 2012). However, how these needs are expressed through social media activities is not clear. Also, how these activities can be related to flourishing in adolescence is still unexplored.

A recent systematic review (Gudka et al., 2023) summarized 118 studies on social media use using a framework based on flourishing dimensions. Although the focus was not on adolescents, it can be a starting point to delineate which social media activities contribute to enhancing well-being. First, and according to the SDT’s need for relatedness, studies showed that social media use augmented social support and belongingness and diminished the sense of loneliness, especially in people with specific needs and minorities. Similarly, social media fostered social capital, including both weak and strong ties, bridging and bonding capital, thus augmenting emotional support and networking value. Nevertheless, social support and enhancement of well-being offered by social media were short (Marciano et al., 2022c) and particularly useful for momentary emotional relief from stressful situations (Neubaum et al., 2014; Bayer et al., 2018). Indeed, according to the interpersonal-connection-behaviors framework (Clark et al., 2018), the quality of the social connection experienced through social media depends on the capacity of the user to build meaningful social connections or not. Similarly, the Internet-enhanced self-disclosure hypothesis and the Evolutionary mismatch model propose two opposite views through which social connections would improve vs. diminish well-being *via* the quality of online relationships (Marciano et al., 2022d). According to a study on 872 Chinese adolescents, active social media use positively influenced adolescents’ flourishing, but this effect was mediated by online and offline social capital (Liu et al., 2020), thus suggesting how social capital has evolved in two forms today. Nevertheless, according to the authors, “the online environment still needs to find a foothold in offline life to influence individuals” (p. 6).

Second, according to the SDT’s need for autonomy, social media use can be beneficial since it enables authentic self-expression and the process of narrative identity (McAdams, 2011), described as the internalized and meaningful story of the evolving self. Authentic self-expression and authenticity on social media lead to higher subjective well-being, including a positive mood and affect (Bailey et al., 2020). In this regard, self-disclosure on social media could facilitate building a sense of autonomy and identity development while, at the same time, maintaining close relationships with others (McLean et al., 2010). For example, narrative identities facilitate young people to build a coherent sense of themselves from the life experiences shared online (Gudka et al., 2023). Narratives are tools to develop identity by reflecting on past experiences in relation to the present and future self (Habermas and Bluck, 2000). At the same time, individual identity is socially construed, especially during the adolescent period. Indeed, a healthy identity exploration includes developing an independent sense of self, while in the context of close relationships, a process called “individuation” (McLean et al., 2010). Thus, social media might allow the integration of others’ feedback in their identity formation as well as values and behaviors to create a sense of social identity (Tajfel and Turner, 2004).

Third, social media may enhance the need for competence through the exploration of one’s interests. Interacting with digital tools expands existing abilities and skills (e.g., creativity through content creation; Rasheed et al., 2020). In particular, social media use fosters positive emotions through the exploration of personal interests and the discovery of new areas of learning, including topics such as cooking, sports, learning about other cultures, and being exposed to new ideas and inspirational contents (Weinstein, 2018). In other words, “interest-driven exploration” on social media refers to an active search for inspiration (Weinstein, 2018). In this regard, positive envy experienced on social media can drive inspiration through assimilation (Meier and Schäfer, 2018), i.e., when the individual focuses on how to become similar to the (upward) comparison target. By doing so, virtual communities that encourage, inspire, and share success would lead to a high sense of self-worth and accomplishment (de la Peña and Quintanilla, 2015; Meier and Schäfer, 2018; Rieger and Klimmt, 2019a). Hence, social media can be seen as a source of “daily doses” of inspiration by receiving and scrolling “eudaimonic messages,” for example, through posts and memes (Rieger and Klimmt, 2019a). Interestingly, social media-based eudaimonic messages have been described as practical, i.e., they refer to situations and tasks people face daily, like motivating oneself to study for an exam or going to the gym despite being tired, thus reflecting how the presence of social media is pervasive and rooted in users’ daily experiences. Also, social media-based eudaimonic content includes visual beauty and pleasantness posts thus offering short moments of contemplation and promoting mindfulness experiences (Rieger and Klimmt, 2019a,b).

Due to the paucity of literature on flourishing and social media use in adolescents, the present study aims to investigate which social media experiences are related to flourishing. In particular, guided by the developmental needs pointed out by the SDT, we included positive and negative social media experiences, self-disclosure on social media, and social media inspiration as proxies of the online fulfillment of SDT needs, to investigate how they were associated with flourishing. We aim to estimate the associations of social media activities with flourishing over and above confounding variables, including other conceptualizations of well-being (i.e., self-esteem), ill-being (e.g., psychopathological symptoms), personality (extraversion and neuroticism), perceived stress, perceived school environment, and self-reported level of physical activity, and sociodemographic factors.

## Methods

### Study design and sample

Data were collected from Swiss adolescents during the first wave of the longitudinal “HappyB” project,^1^ carried out in Spring 2022, in canton Ticino, Italian-speaking Switzerland. Four high schools located in different regions were involved for a total of 1,662 students and 79 classes in the first and second high school years. Data were collected through an online questionnaire *via* Qualtrics during a school hour with the presence of a teacher previously trained by the research team. Students were invited to participate through a flyer distributed in classrooms, including information about the study’s aim. According to the Swiss ethics guidelines, adolescent participants aged 14 and over can provide consent by themselves if the study entails minimal risks. Hence, participants gave their consent directly at the beginning of the online questionnaire. Of the initial sample, 145 participants were not present on the day of data collection or decided not to participate in the study, 28 did not complete the questionnaire, and 57 reported an invalid answer to the control question (i.e., “To check your attention, we ask you to select the number 3 from the list”). Three participants were outliers as they had z-scores > |3.5| (Iglewicz and Hoaglin, 1993) in the included variables, hence they were excluded from the analysis. The study was approved by the regional education administration and by the ethics committee of USI Università della Svizzera italiana, Lugano (Switzerland).

### Measures

Measures were translated from English into Italian when necessary and independently back-translated to assure linguistic validity. A complete list of items and response options is reported in Supplementary Table 1. Descriptive statistics for each included concept are summarized in Table 1. To note, measures used to investigate SDT constructs are proxies of relatedness, autonomy, and competence in the context of social media use in adolescents.

**Table 1:** Sample characteristics (*N* = 1,429) and variables’ descriptive information.

Sample characteristics	M or n		SD or %	
Females	840		58.8	
Males	589		41.2	
First high school year	788		55.1	
Second high school year	641		44.9	
Age	15.84		0.83	
Perceived SES	2.00		0.814	
**Variables**	**M**	**SD**	**Median**	**Range**
Flourishing	7.22	1.47	7.49	2.17–10
Positive social media experiences	5	1.28	5.2	1–7
Negative social media experiences	3.55	1.43	3.75	1–7
Self-disclosure on social media (with close friends)	2.84	0.77	3	1–4
Self-disclosure on social media (with acquaintances or famous people)	1.93	0.75	2	1–4
Social media inspiration (from others)	5.57	2.25	5.7	0–10
Social media inspiration (for others)	4.84	2.49	5.1	0–10
Self-esteem	2.73	0.88	2.62	1–5
Ill-being	2.77	0.59	2.8	1–4
Extraversion	5.13	2.21	5.05	0–10
Neuroticism	5.36	2.63	5.32	0–10
Perceived school environment	3.16	0.718	3	1–4
Self-reported level of physical activity	6.2	2.99	7	0–10
Perceived stress	5.66	2.83	6	0–10

SES, socio-economic status.

#### Outcome

##### Flourishing

It was measured using the Harvard Adolescent Flourishing Measure (ages 12–18; 48). The scale includes 12 items and response options were on a scale from 0 to 10, where higher values indicate higher levels of flourishing (M = 7.22, SD = 1.47, α = 0.840). The following dimensions were investigated: life satisfaction and happiness (2 items), mental and physical health (2 items), meaning and purpose (2 items), character and virtue (2 items), close social relationships (2 items), and financial and material stability (2 items). The confirmatory factor analysis of the Flourishing scale showed good fit indices for the one-factor dimension (χ^2^ = 398.387, df = 53, *p* < 0.001, CFI = 0.914, RMSEA=. 068, SRMR = 0.046; see Supplementary Table 2). In particular, according to the unidimensional factor structure, the item mainly correlated to flourishing in adolescents was the experience of happiness.

#### Key predictors

##### Positive and negative social experiences on social media

Experiences elicited by social media use were measured using nine items from the Online Social Experience Measure (Kent de Grey et al., 2019). The original measure includes 20 items for positive online social experiences (OSEM+ subscale) and 20 for negative online social experiences (OSEM-subscale). We selected a subset of items from the original scale based on their factor loadings. In particular, we included five positive (e.g., “There are people who have faith in me and my abilities.,” “When I feel lonely, there are several people I can talk to”) and four negative online social experiences (e.g., “People have little regard for my emotions.,” “I felt ignored or unimportant to others.”). Answer options ranged from 1 “totally disagree” to 7 “totally agree.” The items were averaged to form a single measure of positive (M_positive_ = 5.00, SD_positive_ = 1.28, α_positive_ = 0.813) and negative (M_negative_ = 3.55, SD_negative_ = 1.43, α_negative_ = 0.802) experiences on social media.

##### Self-disclosure on social media

Two items were used to measure self-disclosure on social media: “I am honest when I express something about myself “and “I express my mood and my feelings*”* (Gibbs et al., 2006). The two statements were repeated for measuring two predictors: (i) self-disclosure to close friends (e.g., best friends, those you interact with every day) and (ii) self-disclosure with acquaintances or famous people (e.g., those people you know “by sight” or bloggers, Youtubers, influencers; Gibbs et al., 2006). Answer options ranged from 1 “never” to 4 “always.” The two items were averaged to form a single measure of self-disclosure with close friends (M = 2.84, SD = 0.77, r = 0.376, *p* < 0.001) and self-disclosure with acquaintances or famous people (M = 1.93, SD = 0.75, r = 0.332, *p* < 0.001).

##### Social media inspiration

It was measured by including four items adapted from other studies looking at positive envy and inspiration (Thrash and Elliot, 2003; Lange and Crusius, 2015). The four items measured two predictors: (i) inspiration from others and (ii) being an inspiration for others. Inspiration for others included the following statement (Thrash and Elliot, 2003; Lange and Crusius, 2015): “On social media, if I notice that a person is better than me, I would try to…” followed by the following four items: ameliorate myself, focus on how to become equally successful, strive to reach the same achievements, and feel inspired. Answer options ranged from 0 “never” to 10 “always.” The four items were averaged to form a single measure of inspiration from others (M = 5.57, SD = 2.25, α = 0.842). Being an inspiration for others included the following statement “On social media, if I notice that I am better at something than others, I would try to…” followed by the following four items: help others to improve, focus on how to help others to be successful in the future in the same way, strive to help others achieve the same goals, and feeling to be an inspiration for others. The four items were averaged to form a single measure of being an inspiration for others (M = 4.84, SD = 2.49, α = 0.880).

#### Control variables

##### Self-esteem

It was measured with the 10-item Rosenberg Self-esteem scale (Rosenberg, 1979; Prezza et al., 1997), with answer options ranging from 1 “completely disagree” to 4 “completely agree.” The items were averaged to form a single measure of self-esteem (M = 2.73, SD = 0.59, α = 0.885).

##### Ill-being

It was measured with eight items adapted from the DSM-5 Self-Rated Level 1 Cross-Cutting Symptom Measure for Child Age 11–17 (Bastiaens and Galus, 2018). The list included the following symptoms experienced in the past month: somatic symptoms (1 item), inattention (2 items), sleep problems (1 item), anxiety (2 items), and depressive symptoms (2 items). Response options ranged from 1 to 5, where 1 “never (none),” 2 “rarely (slight),” 3 “several days (mild),” 4 “more than a half of days (moderate),” and 5 “almost every day (severe).” The items were averaged to form a single indicator of ill-being (M = 2.77, SD = 0.59, α = 0.859).

##### Personality

Personality traits included extraversion and neuroticism, and were measured with four items from the 10-item Big Five Inventory (Guido et al., 2015). Response options ranged from 0 “totally disagree” to 10 “totally agree.” In particular, extraversion were measured by asking participants if they were “outgoing, sociable” and “reserved (reversed item).” The two items were averaged to form a single measure of extraversion (r = 0.352, *p* < 0.001, M = 5.13, SD = 2.21). Neuroticism was assessed by asking if they “get nervous easily” and were “relaxed, handles stress very well (reversed item).” The two items were averaged to form a single measure of neuroticism (r = 0.527, p < 0.001, M = 5.36, SD = 2.63).

##### Other control variables

Other control variables included gender (0 “male,” 1 “female”), perceived socioeconomic status (SES) measured with the following item: “How much wealthy do you think your family is?” (from 1 “Definitely wealthy” to 5 “Definitely not wealthy”), high school year attended (0 “first year,” 1 “second year”), perceived school environment with the following item “In general, do you feel comfortable in your high school?” (from 1 “Not at all” to 4 “A lot”), self-reported level of physical activity with the item “How much do you consider yourself a sporty person?” (from 0 “not at all” to 10 “very much”), and perceived stress with the item “During the last month, what was your daily stress level? (from 0 “not stressed at all” to 10 “very stressed”).

### Analytical plan

After checking that variables were normally distributed (skewness and kurtosis < |1|), we computed Pearson’s correlations among all the included variables with the Flourishing scale. Then, we ran a hierarchical regression analysis with flourishing levels as the outcome. At each step, we added a different set of control variables and social media activities. In particular, in Model 1 we included gender, perceived SES, high school year attended, and age; in Model 2, we further added perceived school environment, self-reported level of physical activity, perceived stress, self-esteem, ill-being, and personality as control variables; in Model 3, we additional included positive and negative online social experiences, self-disclosure on social media, and social media inspiration as predictors. We controlled for multicollinearity (VIF > 5) and applied a Bonferroni’s correction to interpret the results. Missing data were excluded listwise (<1.3% for all the variables). Additionally, we further explored which online social media activities were most related to flourishing with an additional regression analysis.

## Results

The final analytical sample was composed of 1,429 participants (86%) attending the first (n = 788, 55.1%) and second (n = 641, 44.9%) high school year, with a mean age of 15.84 years (SD = 0.83). Around half of the sample was composed of females (n = 840, 58.8%) and most reported being wealthy or very wealthy (n = 1,070, 76.4%). Table 1 shows sample characteristics and variables’ descriptive information.

Pearson’s correlations showed that flourishing levels correlated with all the included variables (see Table 3). In order, flourishing positively correlated with self-esteem (r = 0.651, *p* < 0.001), extraversion (r = 0.276, *p* < 0.001), self-reported level of physical activity (r = 0.325, *p* < 0.001), perception of the school environment (r = 0.391, *p* < 0.001), positive social media experiences (r = 0.314, *p* < 0.001), self-disclosure on social media with close friends (r = 0.206, *p* < 0.001), social media inspiration from others (r = 0.171 *p* < 0.001) and for others (r = 0.167, *p* < 0.001), and self-disclosure on social media with acquaintances and famous people (r = 0.129, *p* < 0.001). Conversely, flourishing negatively correlated with ill-being (r = −0.569, *p* < 0.001) and neuroticism (r = −0.343, *p* < 0.001), negative social media experiences (r = −0.417, *p* < 0.001), perceived stress (r = −0.356, *p* < 0.001), low perceived SES (r = −0.213, *p* < 0.001), female gender (r = −0.189, *p* < 0.001), attending the second high school year (r = −0.084, *p* < 0.001), and age (r = −109, *p* < 0.001; Table 2).

**Table 2:** Correlations table of all the variables included in the regression models.

	1.	2.	3.	4.	5.	6.	7.	8.	9.	10.	11.	12.	13.	14.	15.	16.	17.
Flourishing	1																
Positive social media experiences	0.314^**^	1															
Negative social media experiences	−0.417^**^	−0.149^**^	1														
Self-disclosure on social media (with close friends)	0.206^**^	0.353^**^	−0.102^**^	1													
Self-disclosure on social media (with acquaintances or famous people)	0.129^**^	0.183^**^	−0.080^**^	0.376^**^	1												
Social media inspiration (from others)	0.171^**^	0.174^**^	0.025	0.128^**^	0.114^**^	1											
Social media inspiration (for others)	0.167^**^	0.268^**^	−0.032	0.169^**^	0.185^**^	0.399^**^	1										
Self-esteem	0.651^**^	0.225^**^	−0.482^**^	0.147^**^	0.122^**^	0.089^**^	0.063^*^	1									
Ill-being	−0.569^**^	−0.101^**^	0.470^**^	−0.052	−0.052^*^	0.001	0.008	−0.630^**^	1								
Extraversion	0.276^**^	0.208**	−0.204^**^	0.169^**^	0.128^**^	0.087^**^	0.099^**^	0.297^**^	−0.209^**^	1							
Neuroticism	−0.343^**^	−0.043	0.361^**^	−0.044	−0.066*	0.004	0.027	−0.500^**^	0.536^**^	−0.160^**^	1						
Perceived school environment	0.391^**^	0.159^**^	−0.241^**^	0.072^**^	0.068*	0.050	0.062*	0.332^**^	−0.341^**^	0.138^**^	−0.220^**^	1					
Self-reported level of physical activity	0.325^**^	0.108^**^	−0.136^**^	0.052	0.093^**^	0.139^**^	0.058^*^	0.300^**^	−0.251^**^	0.206^**^	−0.190^**^	0.111^**^	1				
Perceived stress	−0.356^**^	−0.017	0.311^**^	−0.002	−0.011	0.033	0.031	−0.479^**^	0.623^**^	−0.154^**^	0.588^**^	−0.286^**^	−0.119^**^	1			
Gender (1 = female)	−0.189^**^	0.046	0.208^**^	0.051	−0.014	−0.032	0.119^**^	−0.316^**^	0.349^**^	−0.093^**^	0.373^**^	−0.062^*^	−0.283^**^	0.338^**^	1		
Perceived SES	−0.213^**^	−0.095^**^	0.133^**^	−0.014	0.005	−0.027	0.003	−0.182^**^	0.144^**^	−0.088^**^	0.051	−0.120^**^	−0.147^**^	0.050	0.042	1	
High school year (1 = second year)	−0.084^**^	−0.019	0.046	0.004	0.034	−0.083^**^	−0.100^**^	−0.063^*^	0.120^**^	0.001	0.056^*^	−0.043	−0.035	0.101^**^	−0.008	0.008	1
Age	−0.109^**^	−0.003	0.043	−0.017	0.049	−0.033	−0.039	−0.086^**^	0.124^**^	0.009	0.055^*^	−0.093^**^	−0.073^**^	0.087^**^	−0.056^*^	0.104^**^	0.658^**^

^*^p < 0.05, ^**^p < 0.01. SD, Self-disclosure.

**Table 3:** Hierarchical regression results with flourishing as outcome.

	Model 1				Model 2				Model 3			
	B	(SE)	Beta	*p*	B	(SE)	Beta	*p*	B	SE	Beta	*p*
(Constant)	10.559	0.876		0.000	3.752	0.717		0.000	3.836	0.701		0.000
Gender (1 = female)	−0.566	0.077	−0.189	0.000	0.175	0.064	0.058	0.006	0.113	0.063	0.038	0.071
Perceived SES	−0.362	0.047	−0.200	0.000	−0.117	0.035	−0.064	0.001	−0.106	0.034	−0.059	0.002
High school year (1 = second year)	−0.085	0.101	−0.029	0.400	−0.054	0.074	−0.018	0.463	0.016	0.072	0.005	0.821
Age	−0.136	0.061	−0.077	0.026	0.006	0.045	0.003	0.902	−0.020	0.043	−0.011	0.641
Perceived school environment					0.328	0.042	0.160	0.000	0.283	0.040	0.138	0.000
Self-reported level of physical activity					0.061	0.010	0.123	0.000	0.054	0.010	0.109	0.000
Perceived stress					0.022	0.014	0.042	0.113	0.009	0.013	0.017	0.513
Self-esteem					1.043	0.065	0.418	0.000	0.874	0.065	0.350	0.000
Ill-being					−0.433	0.047	−0.261	0.000	−0.418	0.046	−0.252	0.000
Extraversion					0.047	0.013	0.070	0.000	0.023	0.013	0.035	0.074
Neuroticism					0.020	0.014	0.035	0.157	0.020	0.013	0.036	0.131
Positive social media experiences									0.129	0.023	0.112	0.000
Negative social media experiences									−0.079	0.022	−0.076	0.000
SD (with close friends)									0.111	0.040	0.057	0.005
SD (with acquaintances or famous people)									−0.035	0.039	−0.018	0.365
Social media inspiration (from others)									0.047	0.013	0.072	0.000
Social media inspiration (for others)									0.036	0.012	0.060	0.003
Adjusted R square	0.087^**^				0.515^**^				0.553^**^			

B, Unstandardized beta; SE, standard error; β, Standardized beta; *p*, value of *p*; SES, socio-economic status; SD, self-disclosure. **p < 0.01.

Results of the hierarchical regression analysis explained more than half of the variance in the Flourishing scale (R^2^ = 0.553). After applying Bonferroni’s correction (*p* < 0.003) and controlling for all covariates, the following social media activities positively predicted flourishing: positive social media experiences (β = 0.112, *p* < 0.001) and social media inspirations from others (β = 0.072, p < 0.001) and partially for others (β = 0.060, *p* = 0.003). Self-disclosure on social media with close friends (β = 0.057, *p* = 0.005) and with acquaintances or famous people (β = −0.018, *p* = 0.365) were not significant predictors. Conversely, flourishing levels were inversely associated with negative social media experiences (β = −0.076, *p* < 0.001). Among covariates, self-esteem (β = 0.350, *p* < 0.001), ill-being (β = −0.252, *p* < 0.001), perceived school environment (β = 0.138, *p* < 0.001), self-reported level of physical activity (β =0.109, *p* < 0.001), and perceived SES (β = −0.059, *p* = 0.001) were all related to flourishing. Whereas gender, high school year attended, age, personality, and perceived stress showed no significant associations with the outcome.

A more details analysis using the items of social media activities influencing flourishing (i.e., positive and negative social media experiences and social media inspiration for others, see Supplementary Table 3) showed that, after applying Bonferroni’s correction (*p* < 0.004), the positive social media experiences significantly and positively associated with flourishing were the possibility of having several people to talk to on social media when someone was feeling lonely (β = 0.146, *p* < 0.001), having people praising accomplishments (β = 0.102, *p* < 0.001), and having people with faith in the person and his/her abilities (β = 0.118, *p* < 0.001). Additionally, feeling inspired (β =0.111, *p* < 0.001) was associated with higher flourishing. Conversely, feeling ignored (β = −0.124, *p* < 0.001), the presence of people with little regard for emotions (−0.064, *p* < 0.001), and feeling excluded (β = −0.056, *p* < 0.001) were negatively related to flourishing.

## Discussion

In the current study, we explored social media use in adolescents using a positive well-being framework. In particular, drawing from cross-sectional data collected in a large sample of adolescents, we investigated how different social media experiences were associated with flourishing. The concept of flourishing has already been defined by VanderWeele (2017) and VanderWeele et al. (2019), but research on adolescents’ flourishing is scarce, and, to our knowledge, no study has explored its relationship with different social media experiences. In this paper, we aimed to explore how adolescents’ social media use influences flourishing guided by the three pathways pointed out by the SDT: augmenting or diminishing relatedness through positive or negative online social experiences, fostering autonomy through self-disclosure, and augmenting competence through inspirational contents. To control for confounding variables, we included self-esteem, ill-being, and personality (extraversion and neuroticism) as covariates and other socio-demographics and contextual factors. Results showed the following three major findings.

First, among all the social media activities, flourishing was mainly related to positive social media experiences. In particular, some positive social media experiences showed moderate and positive associations with the outcome. For example, the possibility of having several people to talk to when someone is feeling lonely was the social media experience mostly related to flourishing in adolescents. This positive association was present over and above control variables such as self-esteem, ill-being, and personality. This result is in line with a recent meta-analysis showing that good social connections are an important protective factor against ill-being in adolescence (Rueger et al., 2016), although the source of social connection changes from family to peers during the adolescent years. In general, research in diverse disciplines demonstrated that social connections are crucial for well-being and physical health (Morina et al., 2021), including stress management (Gunnar and Hostinar, 2015). Having a large network of peer relationships that are perceived as supportive acts as a safe and predictable environment improving rewarding experiences (Durlak et al., 2011) and offering an effective buffer against negative emotions (Gariépy et al., 2016). One widely explored pathway through which social connection fosters well-being is the role of social support in stressful situations (Holt-Lunstad, 2022), e.g., in a moment of loneliness. This result is crucial, considering that a study including data on 37 countries showed that loneliness rates in adolescents increased after 2012 “in conjunction with the rise of smartphone access and increased internet use, though causation cannot be proven” (Gunnar and Hostinar, 2015, p.257). Indeed, in disentangling between- vs. within-person effects, Twenge et al. (2019) found that, at the within-person level, young people using social media more often than their own average levels of use also accrued social capital and opportunities for in-person interactions, thus experiencing less loneliness. In addition to this result, relationship quality is crucial. Supportive and receptive peers promote positive affect, whereas adolescents who tend to co-ruminate on negative outcomes have protracted negative feelings (Stewart et al., 2013). Indeed, not surprisingly, in our study, negative social media experiences like feeling ignored, excluded, and interacting with people having little or no regard for participants’ emotions were associated with lower levels of flourishing. Notably, the negative effects of social relations could be even worse in adolescence since the developing brain is particularly susceptible to others’ behaviors and judgments (Blakemore, 2008; Somerville, 2013), and affective consequences of negative social experiences are greater in adolescents compared to adulthood (Sebastian et al., 2010). This aligns with a study on 9,107 adolescents showing that happiness was primarily and positively associated with good social connections (e.g., with family, friends, and school), and it was negatively related to bullying behaviors and discrimination (Lambert et al., 2014). Furthermore, flourishing was positively correlated with self-disclosure on social media. However, the association was not significant in the regression analysis. Probably, the measure of self-disclosure showed some overlaps with the construct of positive online social experiences, although there was no collinearity between the two variables. In particular, it is challenging to disentangle self-disclosure from social connection since the two are interrelated: indeed, the development of identity and autonomy happens in the context of close relationships (McLean et al., 2010).

Second, our results showed that inspiration through social media content (i.e., being inspired by others’ posts and activities and being an inspiration for others) improves flourishing levels in adolescents. Positive affective outcomes, including flourishing, might be fostered by pursuing social media posts and content related to personal activities and hobbies (Meier and Schäfer, 2018). Indeed, the exploration of one’s interests and the discovery of new areas of learning relieve distress and allow for spreading positive messages (Weinstein, 2018). Active posting and engaging with hedonic or inspiring social media content has improved well-being, including increasing love and compassion toward others (Janicke-Bowles et al., 2022). Also, inspiration shapes how people share: inspiring messages are more likely to be shared in new ways by the users (Xia and Wai Li, 2022). Inspirational moments on social media have also been described as gratifying (Rieger and Klimmt, 2019a), with the potential to match users’ situational demands with meaning, thus giving small drives of mindfulness and psychological healing in daily life (Rieger and Klimmt, 2019a). Finally, well-being can be fostered through the assimilation of upward comparison targets through benign envy (Meier and Schäfer, 2018). For example, evocative objects of inspiration may offer new or better possibilities, thus motivating a person to take action and ameliorate oneself (Meier et al., 2020), thus contributing to different facets of well-being, like hedonic and eudaimonic, both tackled by flourishing sub-dimensions.

Third, different covariates showed moderate to strong associations with flourishing. First, self-esteem was largely and positively associated, whereas ill-being was largely and negatively associated with flourishing. This result does not surprise, considering that flourishing can be seen as the extreme of a continuum, where ill-being is the opposite side. However, well-being does not reflect the absence of ill-being (Zhao and Tay, 2022): the mid-point can be defined as a “neutral and nominal zero” (Janicke-Bowles et al., 2022, p. 3). Additionally, a good school environment (e.g., with peers and teachers) was positively associated with flourishing. This result reflects that flourishing encompasses different dimensions, including socio-ecological factors (Kim et al., 2020), and each of them should be considered in the evaluation of adolescents’ well-being. In general, positive emotions during school are associated with more student engagement and adaptive coping in students (Reschly et al., 2008), and interventions based on positive psychology showed that participants improved positive emotions and increased levels of calmness and enjoyment (Laakso et al., 2022). Also, flourishing has been related to supportive school environments, students’ sense of belonging to the school, and civic engagement (Fink, 2014), thus underlying the importance of considering the adolescent’s environment when exploring well-being. Additionally, in line with other studies focusing on well-being outcomes such as life satisfaction, flourishing levels were related to being physically active, carrying out vigorous exercises, and participating in group sports (Proctor et al., 2009). Also, a meta-analysis reported the beneficial effects of physical activity interventions on well-being outcomes including resilience and positive mental health (Andermo et al., 2020) as well as health-related quality of life (Marker et al., 2018). Furthermore, a lower perceived SES was negatively related to flourishing. This negative association could be partly due to the fact that flourishing encompasses financial stability. Also, according to the social determinants of health, health outcomes differ by socioeconomic factors and racial and ethnic characteristics (Fernandez and Kulik, 1981; Huebner, 1991; Verme, 2011). Health inequities lead to differences in motivations, access, and engagement with communication, such as social media (Viswanath and Emmons, 2006; Kontos et al., 2007; Bekalu et al., 2019; Viswanath et al., 2021). Finally, extraversion—and not neuroticism—was related to flourishing but not in the final regression model. This result aligns with extroverts’ tendency to display positivity and use social media frequently to maintain offline social contacts and create content (Amichai-Hamburger et al., 2002). On the contrary, neuroticism has been related to problematic digital media use and addictive online behaviors (Marciano et al., 2020).

To conclude, good habits encompass different dimensions of youth’s well-being and can act as a “mental vaccine” enhancing brain resilience (Ekman et al., 2022). Thus, stemming from the results of our study, we suggest that, together with a range of good habits, including “healthy eating, exercising, rest and sleep, optimism, managing stress, making autonomous decisions, variety and challenge, social interactions with friends, learning new things, and repetition” (Twenge et al., 2019, p. 13), also using social media in a way that promotes rather than diminish well-being should thus be included as an additional good habit. This would further diminish the gap between public health research and public health practice (Colditz et al., 2008). Overall, these positive habits would likely influence the development of molecules crucial for good brain development and influence genes and brain plasticity, hopefully in a way that would promote positive affect and behavioral outcomes (Ekman et al., 2022).

## Limitations and future directions

Some methodological limitations should be acknowledged. First, due to the cross-sectional design of our study, we cannot draw any conclusions on the causes and effects of flourishing. In this regard, we cannot exclude the reverse associations and directionality, that is, flourishing can predict a specific use of social media. Indeed, it is somewhat possible that flourishing causes productive and positive uses of social media, as well as previously shown in other studies focusing on ill-being outcomes (Marciano et al., 2022d), although the effect from ill-being to media use was stronger. Also, we included items as proxies for SDT constructs. Hence future studies should include direct measures of relatedness, autonomy, and competence that have been developed and adapt them to the social media context. Although we considered personality traits such as extraversion and neuroticism as covariates, future studies should consider all Big Five personality traits and look at differences between high vs. low levels of such traits. Additionally, we did not measure the extent of social media use. Hence, we cannot exclude that the relationship between social media time and flourishing would be moderated by the quality and type of one’s experience. Although our study makes a step forward in the discussion of positive and negative media effects, it does not completely resolve the current debate.

Although we found significant associations between adolescents’ socio-contextual factors and flourishing levels, we did not include questions related to parenting or other family factors, like the quality of the parent–child relationship, which might further determine adolescents’ well-being (Kim et al., 2020). Also, we measured self-reported physical activity with one item as a reliable proxy of objective levels (Scott et al., 2015). However, we suggest future studies include more reliable measures, such as the use of accelerometers and wearable devices. Although we included control variables such as self-esteem and ill-being, future studies should consider other (well-being) outcomes such as hedonic and eudaimonic well-being. Additionally, our sample might not be representative of vulnerable adolescents and minorities, hence the study results should be replicated in other cultural and contextual settings. The drop-out of participants might have been due to specific suffering conditions or reasons other than the ones listed. Furthermore, results represent a specific cohort of students (i.e., first and second high school year) and do not extend to other age groups showing different social media usages (Orben et al., 2022) or students attending different educational paths in Switzerland, like apprenticeships.

Eventually, future studies should further provide longitudinal insight into the protective and risk factors influencing flourishing. Also, they should focus more on the dose–response mechanism of social media use and well-being and disentangle between- vs. within-person dynamics, using intensive longitudinal data and investigating the temporal dynamics (e.g., how long does a positive effect of social media use last?; Marciano et al., 2022c). Furthermore, since we did not assess other variables related to a social determinant approach to health outcomes (Koh et al., 2010), additional control measures should include a more detailed evaluation of socioeconomic status. For example, further studies should focus on digital inequality, referring to how people’s societal position affects their digital access, skills, and types of uses, as well as the outcomes of digital engagement, ultimately feeding back into their life chances. Although researchers examined the relationship between social media use and well-being and the role of social inequality (Buchi and Hargittai, 2022), more work should be done on flourishing. For example, Skogen et al. (2022) investigated the association between subjective SES, frequency, and daily duration of social media use and self-reported negative experiences of social media platforms in high school students in Norway. They found consistent and strong support for an association between SES and negative experiences on social media, even after adjusting for the amount of social media use. That said, flourishing is a much broader field, as mentioned above, and to better determine flourishing, over time, we might need to assess financial stability in various domains and not be limited to SES only (VanderWeele, 2017).

## Conclusion

Increasing attention has been given to *ill*- instead of *well*-being indicators like flourishing (VanderWeele et al., 2020). Using well-being measures to investigate the link between social media use and well-being in adolescents is a promising tool that allows moving forward the ill-being framework. Results showed that positive online social media activities, together with the feeling of inspiration through social media, fostered flourishing, whereas negative online social media experiences showed the opposite association. These relationships were present even after controlling for covariates such as self-esteem, mental health problems, and personality. Results aligned with the SDT and suggested that specific social media activities promote well-being and, thus, should be additionally included as a good habit influencing adolescents’ development. In particular, since 40 to 80% of health and wellness can be somehow attributed to social factors (Hood et al., 2016), improving social relationships might become a priority in the current public health agenda (Holt-Lunstad et al., 2017) both in the United States and globally (Holt-Lunstad, 2022). Thus, interventions aiming to foster adolescents’ flourishing should include curricula aiming to improve a good use of social media through positive online relationships and the consumption of inspiring contents.

## Data availability statement

The datasets presented in this study can be found in online repositories. The names of the repository/repositories and accession number(s) can be found at: https://osf.io/k6xpw.

## Ethics statement

The studies involving human participants were reviewed and approved by USI Università della Svizzera italiana. Written informed consent from the participants’ legal guardian/next of kin was not required to participate in this study in accordance with the national legislation and the institutional requirements.

## Author contributions

LM conceived the project, collected the data, provided funding, performed the statistical analysis, and wrote the first draft of the manuscript. KV wrote sections of the manuscript. All authors contributed to the article and approved the submitted version.

## Funding

The study was funded by the Swiss National Science Foundation (grant no. P500PS_202974).

## Conflict of interest

The authors declare that the research was conducted in the absence of any commercial or financial relationships that could be construed as a potential conflict of interest.

## Publisher’s note

All claims expressed in this article are solely those of the authors and do not necessarily represent those of their affiliated organizations, or those of the publisher, the editors and the reviewers. Any product that may be evaluated in this article, or claim that may be made by its manufacturer, is not guaranteed or endorsed by the publisher.

## Footnotes

^1^ https://mediaticino.usi.ch/en/happyb

## References

[ref1] Amichai-Hamburger Y. Wainapel G. Fox S. (2002). On the internet no one knows I’m an introvert: Extroversion, Neuroticism, and Internet Interaction. Cyberpsychol. Behav. 5, 125–128. doi: 10.1089/109493102753770507, PMID: 12025878

[ref2] Andermo S. Hallgren M. Nguyen T. T. D. Jonsson S. Petersen S. Friberg M.. (2020). School-related physical activity interventions and mental health among children: a systematic review and meta-analysis. Sports Med Open 6, 6:25:25. doi: 10.1186/s40798-020-00254-x, PMID: 32548792 PMC7297899

[ref3] Bailey E. R. Matz S. C. Youyou W. Iyengar S. S. (2020). Authentic self-expression on social media is associated with greater subjective well-being. Nat. Commun. 11:4889. doi: 10.1038/s41467-020-18539-w, PMID: 33024115 PMC7538578

[ref4] Bastiaens L. Galus J. (2018). The DSM-5 self-rated level 1 cross-cutting symptom measure as a screening tool. Psychiatry Q. 89, 111–115. doi: 10.1007/s11126-017-9518-7, PMID: 28523431

[ref5] Bayer J. Ellison N. Schoenebeck S. Brady E. Falk E. B. (2018). Facebook in context(s): measuring emotional responses across time and space. New Media Soc. 20, 1047–1067. doi: 10.1177/1461444816681522

[ref6] Bekalu M. A. RF M. C. Viswanath K. (2019). Association of Social Media use with Social Well-Being, positive mental health, and self-rated health: disentangling routine use from emotional connection to use. Health Educ. Behav. 46, 69S–80S. doi: 10.1177/1090198119863768, PMID: 31742462

[ref7] Blakemore S. J. (2008). The social brain in adolescence. Nat. Rev. Neurosci. 9, 267–277. doi: 10.1038/nrn2353 18354399

[ref8] Booker C. L. Skew A. J. Kelly Y. J. Sacker A. (2015). Media use, sports participation, and well-being in adolescence: cross-sectional findings from the UK household longitudinal study. Am. J. Public Health 105, 173–179. doi: 10.2105/AJPH.2013.301783, PMID: 25494209 PMC4265914

[ref9] Buchi M. Hargittai E. (2022). A need for considering digital inequality when studying social media use and well-being. Soc Media Soc. 8:205630512110691. doi: 10.1177/20563051211069125

[ref10] Camerini A. L. Gerosa T. Marciano L. Predicting problematic smartphone use over time in adolescence: a latent class regression analysis of online and offline activities: New Media Soc (2020) Available at: https://journals.sagepub.com/eprint/VBWRZ9NWEGU7HRXRH7W7/full (Accessed 25 August 2020).

[ref11] Clark J. L. Algoe S. B. Green M. C. (2018). Social network sites and well-being: the role of social connection. Curr. Dir. Psychol. Sci. 27, 32–37. doi: 10.1177/0963721417730833

[ref12] Colditz G. A. Emmons K. M. Vishwanath K. Kerner J. F. (2008). Translating science to practice: community and academic perspectives. J. Public Health Manag. Pract. 14, 144–149. doi: 10.1097/01.PHH.0000311892.73078.8b 18287920

[ref13] de la Peña A. Quintanilla C. (2015). Share, like and achieve: the power of Facebook to reach health-related goals. Int J Consum Stud 39, 495–505. doi: 10.1111/ijcs.12224

[ref14] Dickson K. Richardson M. Kwan I. MacDowall W. Burchett H. Stansfield C.. Screen-based activities and children and young people’s mental health and psychosocial wellbeing: a systematic map of reviews. (2018). Available at: https://researchonline.lshtm.ac.uk/id/eprint/4656950/ (Accessed 11 December 2020). 10.1089/cyber.2019.0370 PMC7044782 31977251

[ref15] Durlak J. A. Weissberg R. P. Dymnicki A. B. Taylor R. D. Schellinger K. B. (2011). The impact of enhancing students’ social and emotional learning: a meta-analysis of school-based universal interventions. Child Dev. 82, 405–432. doi: 10.1111/j.1467-8624.2010.01564.x, PMID: 21291449

[ref16] Ekman R. Fletcher A. Giota J. Eriksson A. Thomas B. Bååthe F. (2022). A flourishing brain in the 21st century: a scoping review of the impact of developing good habits for mind, brain, well-being, and learning. Mind Brain Educ. 16, 13–23. doi: 10.1111/mbe.12305

[ref17] Federal Statistical Office. Health—pocket statistics 2022 | publication. Neuchâtel; (2022). Available at: https://www.bfs.admin.ch/asset/en/21244125

[ref18] Fernandes B. Biswas U. N. Mansukhani R. T. Casarín A. V. Essau C. A. (2020). The impact of COVID-19 lockdown on internet use and escapism in adolescents. Rev Psicol Clínica Con Niños Adolesc 7, 59–65. doi: 10.21134/rpcna.2020.mon.2056

[ref19] Fernandez R. M. Kulik J. C. (1981). A multilevel model of life satisfaction: effects of individual characteristics and neighborhood composition. Am. Sociol. Rev. 46, 840–850.

[ref20] Fink J. E. (2014). Flourishing: exploring predictors of mental health within the college environment. J. Am. Coll. Health 62, 380–388. doi: 10.1080/07448481.2014.917647, PMID: 24779485

[ref21] Gariépy G. Honkaniemi H. Quesnel-Vallée A. (2016). Social support and protection from depression: systematic review of current findings in Western countries. Br. J. Psychiatry 209, 284–293. doi: 10.1192/bjp.bp.115.169094, PMID: 27445355

[ref22] Gibbs J. L. Ellison N. B. Heino R. D. (2006). Self-presentation in online personals: the role of anticipated future interaction, self-disclosure, and perceived success in internet dating. Commun Res 33, 152–177. doi: 10.1177/0093650205285368

[ref23] Gudka M. Gardiner K. Lomas T. (2023). Towards a framework for flourishing through social media: a systematic review of 118 research studies. J. Posit. Psychol. 18:86. doi: 10.1080/17439760.2021.1991447

[ref24] Guido G. Peluso A. M. Capestro M. Miglietta M. (2015). An Italian version of the 10-item big five inventory: an application to hedonic and utilitarian shopping values. Personal. Individ. Differ. 76, 135–140. doi: 10.1016/j.paid.2014.11.053

[ref25] Gunnar M. R. Hostinar C. E. (2015). The social buffering of the hypothalamic-pituitary-adrenocortical axis in humans: developmental and experiential determinants. Soc. Neurosci. 10, 479–488. doi: 10.1080/17470919.2015.1070747, PMID: 26230646 PMC4618716

[ref26] Habermas T. Bluck S. (2000). Getting a life: the emergence of the life story in adolescence. Psychol. Bull. 126, 748–769. doi: 10.1037/0033-2909.126.5.748, PMID: 10989622

[ref27] Henderson L. W. Knight T. (2012). Integrating the hedonic and eudaimonic perspectives to more comprehensively understand wellbeing and pathways to wellbeing. Int J Wellbeing 2, 196–221. doi: 10.5502/ijw.v2i3.3

[ref28] Holt-Lunstad J. (2022). Social connection as a public health issue: the evidence and a systemic framework for prioritizing the “social” in social determinants of health. Annu. Rev. Public Health 43, 193–213. doi: 10.1146/annurev-publhealth-052020-110732 35021021

[ref29] Holt-Lunstad J. Robles T. Sbarra D. A. Advancing social connection as a public health priority in the United States. Am. Psychol., (2017). 72:517–530. /, doi: 10.1037/amp0000103, PMID: 28880099 PMC5598785

[ref30] Hood C. M. Gennuso K. P. Swain G. R. Catlin B. B. (2016). County health rankings: relationships between determinant factors and health outcomes. Am. J. Prev. Med. 50, 129–135. doi: 10.1016/j.amepre.2015.08.024 26526164

[ref31] Huebner E. S. (1991). Correlates of life satisfaction in children. Sch. Psychol. Q. 6, 103–111. doi: 10.1037/h0088805

[ref32] Hui E. K. P. Tsang S. K. M. (2012). Self-determination as a psychological and positive youth development construct. Sci World J 2012:759358, 1–7. doi: 10.1100/2012/759358, PMID: 22649322 PMC3353314

[ref33] Huppert F. A. (2013). Flourishing across Europe: application of a new conceptual framework for defining well-being. Soc Indic Res 110, 837–861. doi: 10.1007/s11205-011-9966-7, PMID: 23329863 PMC3545194

[ref34] Iglewicz B. Hoaglin D. C. How to detect and handle outliers. Milwaukee, Wis: ASQC Quality Press; (1993). 87 p.

[ref35] Janicke-Bowles S. Raney A. Oliver M. Dale K. Zhao D. Neumann D.. (2022). Inspiration on social media: applying an entertainment perspective to longitudinally explore mental health and well-being. Cyberpsychology: Journal of Psychosocial Research on Cyberspace 16. doi: 10.5817/CP2022-2-1

[ref36] Kelly Y. Zilanawala A. Booker C. Sacker A. (2018). Social media use and adolescent mental health: findings from the UK millennium cohort study. EClinicalMed 6, 59–68. doi: 10.1016/j.eclinm.2018.12.005, PMID: 31193561 PMC6537508

[ref37] Kent de Grey R. G. Uchino B. N. Baucom B. R. Smith T. W. Holton A. E. Diener E. F. (2019). Enemies and friends in high-tech places: the development and validation of the online social experiences measure. Digit Health 5, 5:2055207619878351. doi: 10.1177/2055207619878351, PMID: 31579526 PMC6759713

[ref38] Kessler R. C. Amminger G. P. Aguilar-Gaxiola S. Alonso J. Lee S. (2007). ??St??N TB. Age of onset of mental disorders: a review of recent literature. Curr. Opin. Psychiatry 20, 359–364. doi: 10.1097/YCO.0b013e32816ebc8c, PMID: 17551351 PMC1925038

[ref39] Kessler R. C. Berglund P. Demler O. Jin R. Merikangas K. R. Walters E. E. (2005). Lifetime prevalence and age-of-onset distributions of DSM-IV disorders in the National Comorbidity Survey Replication. Arch. Gen. Psychiatry 62, 593–602. doi: 10.1001/archpsyc.62.6.593, PMID: 15939837

[ref40] Kim T. Jang C. Y. Kim M. (2020). Socioecological predictors on psychological flourishing in the US adolescence. Int. J. Environ. Res. Public Health 17:E7917. doi: 10.3390/ijerph17217917 PMC7662773 33126673

[ref41] Koh H. K. Oppenheimer S. C. Massin-Short S. B. Emmons K. M. Geller A. C. Viswanath K. (2010). Translating research evidence into practice to reduce health disparities: a social determinants approach. Am. J. Public Health 100, S72–S80. doi: 10.2105/AJPH.2009.167353, PMID: 20147686 PMC2837437

[ref42] Kontos E. Bennett G. Viswanath K. (2007). Barriers and facilitators to home computer and internet use among urban novice computer users of low socioeconomic position. J. Med. Internet Res. 9:e948. doi: 10.2196/jmir.9.4.e31 PMC2223190 17951215

[ref43] Laakso M. Fagerlund Å. Pesonen A. K. RAO F. Eriksson J. G. (2022). The impact of the positive education program flourishing students on early adolescents’ daily positive and negative emotions using the experience sampling method. J. Early Adolesc. 43, 385–417. doi: 10.1177/02724316221105582

[ref44] Lambert M. Fleming T. Ameratunga S. Robinson E. Crengle S. Sheridan J.. (2014). Looking on the bright side: an assessment of factors associated with adolescents’ happiness. Adv. Ment. Health 12, 101–109. doi: 10.1080/18374905.2014.11081888

[ref45] Lange J. Crusius J. (2015). Dispositional envy revisited: unraveling the motivational dynamics of benign and malicious envy. Pers. Soc. Psychol. Bull. 41, 284–294. doi: 10.1177/0146167214564959 25534243

[ref46] Liu Y. Ni X. Niu G. (2020). The influence of active social networking services use and social capital on flourishing in Chinese adolescents. Child Youth Serv Rev 119:105689. doi: 10.1016/j.childyouth.2020.105689

[ref47] Lomas T. Vanderweele T. J. (2022). The garden and the orchestra: generative metaphors for conceptualizing the complexities of well-being. Int. J. Environ. Res. Public Health 19:14544. doi: 10.3390/ijerph192114544, PMID: 36361423 PMC9657769

[ref48] Marciano L. Camerini A. L. Schulz P. J. (2020). Neuroticism in the digital age: a meta-analysis. Comput Hum Behav Rep 2:100026. doi: 10.1016/j.chbr.2020.100026

[ref49] Marciano L. Driver C. C. Schulz P. J. Camerini A. L. (2022c). Dynamics of adolescents’ smartphone use and well-being are positive but ephemeral. Sci. Rep. 12, 12:1316. doi: 10.1038/s41598-022-05291-y, PMID: 35079056 PMC8789843

[ref50] Marciano L. Ostroumova M. Schulz P. J. Camerini A. L. (2022a). Digital media use and adolescents’ mental health during the COVID-19 pandemic: a systematic review and meta-analysis. Front. Public Health 9:793868. doi: 10.3389/fpubh.2021.793868 35186872 PMC8848548

[ref51] Marciano L. Schulz P. J. Camerini A. L. (2022d). How do depression, duration of internet use and social connection in adolescence influence each other over time? An extension of the RI-CLPM including contextual factors. Comput. Hum. Behav. 136:107390. doi: 10.1016/j.chb.2022.107390

[ref52] Marciano L. Viswanath K. Morese R. Camerini A. L. (2022b). Screen time and adolescents’ mental health before and after the COVID-19 lockdown in Switzerland: a natural experiment. Front. Psych. 13:981881. doi: 10.3389/fpsyt.2022.981881, PMID: 36465307 PMC9709147

[ref53] Marker A. M. Steele R. G. Noser A. E. (2018). Physical activity and health-related quality of life in children and adolescents: a systematic review and meta-analysis. Health Psychol. 37, 893–903. doi: 10.1037/hea0000653 30234348

[ref54] McAdams D. P. (2011). “Narrative identity” in Handbook of identity theory and research. eds. Schwartz S. J. Luyckx K. Vignoles V. L. (New York, NY: Springer), 99–115.

[ref55] McLean K. C. Breen A. V. Fournier M. A. (2010). Constructing the self in early, middle, and late adolescent boys: narrative identity, individuation, and well-being. J Res Adolesc 20, 166–187. doi: 10.1111/j.1532-7795.2009.00633.x

[ref57] Meier A. Gilbert A. Börner S. Possler D. (2020). Instagram inspiration: how upward comparison on social network sites can contribute to well-being. J. Commun. 70, 721–743. doi: 10.1093/joc/jqaa025

[ref58] Meier A. Schäfer S. (2018). The positive side of social comparison on social network sites: how envy can drive inspiration on Instagram. Cyberpsychol. Behav. Soc. Netw. 21, 411–417. doi: 10.1089/cyber.2017.0708, PMID: 29995526

[ref59] Merikangas K. R. Ping H. J. Burstein M. Swanson S. A. Avenevoli S. Cui L.. (2010). Lifetime prevalence of mental disorders in U.S. adolescents: results from the National Comorbidity Survey Replication–Adolescent Supplement (NCS-A). J. Am. Acad. Child Adolesc. Psychiatry 49, 980–989. doi: 10.1016/j.jaac.2010.05.017, PMID: 20855043 PMC2946114

[ref60] Monaco A. P. (2021). An epigenetic, transgenerational model of increased mental health disorders in children, adolescents and young adults. Eur. J. Hum. Genet. 29, 387–395. doi: 10.1038/s41431-020-00726-4, PMID: 32948849 PMC7940651

[ref61] Montag C. Elhai J. D. (2020). Discussing digital technology overuse in children and adolescents during the COVID-19 pandemic and beyond: on the importance of considering affective neuroscience theory. Addict. Behav. Rep. 12:100313. doi: 10.1016/j.abrep.2020.100313 33364321 PMC7752706

[ref62] Morina N. Kip A. Hoppen T. H. Priebe S. Meyer T. (2021). Potential impact of physical distancing on physical and mental health: a rapid narrative umbrella review of meta-analyses on the link between social connection and health. BMJ Open 11:e042335. doi: 10.1136/bmjopen-2020-042335, PMID: 33737424 PMC7978290

[ref63] Neubaum G. Rösner L. Rosenthal-von der Pütten A. M. Krämer N. C. (2014). Psychosocial functions of social media usage in a disaster situation: a multi-methodological approach. Comput. Hum. Behav. 34, 28–38. doi: 10.1016/j.chb.2014.01.021

[ref64] Neufeld SAS (2022). The burden of young people’s mental health conditions in Europe: no cause for complacency. Lancet Reg Health Eur 16:100364. doi: 10.1016/j.lanepe.2022.100364 35345644 PMC8956936

[ref65] Odgers C. L. Jensen M. R. (2020). Annual research review: adolescent mental health in the digital age: facts, fears, and future directions. J. Child Psychol. Psychiatry 61, 336–348. doi: 10.1111/jcpp.13190, PMID: 31951670 PMC8221420

[ref66] Orben A. (2020). Teenagers, screens and social media: a narrative review of reviews and key studies. Soc. Psychiatry Psychiatr. Epidemiol. 55, 407–414. doi: 10.1007/s00127-019-01825-4, PMID: 31925481

[ref67] Orben A. Przybylski A. K. Blakemore S. J. Kievit R. A. (2022). Windows of developmental sensitivity to social media. Nat. Commun. 13:1649. doi: 10.1038/s41467-022-29296-3, PMID: 35347142 PMC8960761

[ref68] Prezza M. Trombaccia F. R. Armento L. (1997). La scala dell’autostima di Rosenberg: Traduzione e validazione Italiana. [The Rosenberg Self-Esteem Scale: Italian translation and validation.]. Giunti Organ Spec. 223, 35–44.

[ref69] Proctor C. L. Linley P. A. Maltby J. (2009). Youth life satisfaction: a review of the literature. J Happiness Stud 10, 583–630. doi: 10.1007/s10902-008-9110-9

[ref70] Przybylski A. K. Weinstein N. (2017). A large-scale test of the goldilocks hypothesis: quantifying the relations between digital-screen use and the mental well-being of adolescents. Psychol. Sci. 28, 204–215. doi: 10.1177/0956797616678438, PMID: 28085574

[ref71] Radtke T. Apel T. Schenkel K. Keller J. von Lindern E. (2022). Digital detox: an effective solution in the smartphone era? A systematic literature review. Mob Media Commun. 10, 190–215. doi: 10.1177/20501579211028647

[ref72] Rasheed M. I. Malik M. J. Pitafi A. H. Iqbal J. Anser M. K. Abbas M. (2020). Usage of social media, student engagement, and creativity: the role of knowledge sharing behavior and cyberbullying. Comput. Educ. 159:104002. doi: 10.1016/j.compedu.2020.104002

[ref73] Reschly A. L. Huebner E. S. Appleton J. J. Antaramian S. (2008). Engagement as flourishing: the contribution of positive emotions and coping to adolescents’ engagement at school and with learning. Psychol Sch. 45, 419–431. doi: 10.1002/pits.20306

[ref74] Rieger D. Klimmt C. (2019a). The daily dose of digital inspiration: a multi-method exploration of meaningful communication in social media. New Media Soc. 21, 97–118. doi: 10.1177/1461444818788323

[ref75] Rieger D. Klimmt C. (2019b). The daily dose of digital inspiration 2: themes and affective user responses to meaningful memes in social media. New Media Soc. 21, 2201–2221. doi: 10.1177/1461444819842875

[ref76] Rosenberg M. Conceiving the self. New York: Basic Books; (1979). 319 p.

[ref77] Rueger S. Y. Malecki C. K. Pyun Y. Aycock C. Coyle S. (2016). A meta-analytic review of the association between perceived social support and depression in childhood and adolescence. Psychol. Bull. 142, 1017–1067. doi: 10.1037/bul0000058, PMID: 27504934

[ref78] Ryan R. M. Deci E. L. (2000). Self-determination theory and the facilitation of intrinsic motivation, social development, and well-being. Am. Psychol. 55, 68–78. doi: 10.1037/0003-066X.55.1.68, PMID: 11392867

[ref79] Ryan R. M. Deci E. L. (2001). On happiness and human potentials: a review of research on hedonic and Eudaimonic well-being. Annu. Rev. Psychol. 52, 141–166. doi: 10.1146/annurev.psych.52.1.141, PMID: 11148302

[ref80] Scott J. J. Morgan P. J. Plotnikoff R. C. Lubans D. R. (2015). Reliability and validity of a single-item physical activity measure for adolescents. J. Paediatr. Child Health 51, 787–793. doi: 10.1111/jpc.12836, PMID: 25643749

[ref81] Sebastian C. Viding E. Williams K. D. Blakemore S. J. (2010). Social brain development and the affective consequences of ostracism in adolescence. Brain Cogn. 72, 134–145. doi: 10.1016/j.bandc.2009.06.008, PMID: 19628323

[ref82] Skogen J. C. Bøe T. Finserås T. R. Sivertsen B. Hella R. T. Hjetland G. J. (2022). Lower subjective socioeconomic status is associated with increased risk of reporting negative experiences on social media. Findings from the “LifeOnSoMe”-study. Front Public Health 10:873463. doi: 10.3389/fpubh.2022.873463, PMID: 35769790 PMC9234458

[ref83] Somerville L. H. (2013). The teenage brain: sensitivity to social evaluation. Curr. Dir. Psychol. Sci. 22, 121–127. doi: 10.1177/0963721413476512, PMID: 24761055 PMC3992953

[ref84] Stewart J. G. Mazurka R. Bond L. Wynne-Edwards K. E. Harkness K. L. (2013). Rumination and impaired cortisol recovery following a social stressor in adolescent depression. J. Abnorm. Child Psychol. 41, 1015–1026. doi: 10.1007/s10802-013-9740-1, PMID: 23553496

[ref85] Süss D. D. Waller G. Jael B. Lilian S. Gregor W. Céline K.. Rapporto sui risultati dello studio JAMES 2020. (2020) 76. Available at: https://www.zhaw.ch/en/psychology/research/media-psychology/media-use/james/#c159120 (Accessed 25 January 2021).

[ref86] Tajfel H. Turner J. C. (2004). “The social identity theory of intergroup behavior,” in Political psychology: Key readings eds. J. T. Jost and J. Sidanius (Psychology Press), 276–293. doi: 10.4324/9780203505984-16

[ref87] Thrash T. M. Elliot A. J. (2003). Inspiration as a psychological construct. J. Pers. Soc. Psychol. 84, 871–889. doi: 10.1037/0022-3514.84.4.871 12703654

[ref88] Twenge J. M. Campbell W. K. (2018). Associations between screen time and lower psychological well-being among children and adolescents: evidence from a population-based study. Prev. Med. Rep. 12, 271–283. doi: 10.1016/j.pmedr.2018.10.003, PMID: 30406005 PMC6214874

[ref89] Twenge J. M. Campbell W. K. (2019). Media use is linked to lower psychological well-being: evidence from three datasets. Psychiatry Q. 90, 311–331. doi: 10.1007/s11126-019-09630-7, PMID: 30859387

[ref90] Twenge J. M. Haidt J. Blake A. B. McAllister C. Lemon H. Le Roy A. (2021). Worldwide increases in adolescent loneliness. J. Adolesc. 93, 257–269. doi: 10.1016/j.adolescence.2021.06.006, PMID: 34294429

[ref91] Twenge J. M. Martin G. N. Campbell W. K. (2018). Decreases in psychological well-being among American adolescents after 2012 and links to screen time during the rise of smartphone technology. Emotion 18, 765–780. doi: 10.1037/emo0000403, PMID: 29355336

[ref92] Twenge J. M. Spitzberg B. H. Campbell W. K. (2019). Less in-person social interaction with peers among U.S. adolescents in the 21st century and links to loneliness. J Soc Pers Relatsh 36, 1892–1913. doi: 10.1177/0265407519836170

[ref93] Valkenburg P. M. Meier A. Beyens I. Social media use and its impact on adolescent mental health: An Umbrella Review of the Evidence. PsyArXiv [Preprint] (2021). Available at: https://psyarxiv.com/y8zdg/ (Accessed 28 July 2021). 10.1016/j.copsyc.2021.08.017 34563980

[ref94] VanderWeele T. J. (2017). On the promotion of human flourishing. Proc Natl Acad Sci 114, 8148–8156. doi: 10.1073/pnas.1702996114 28705870 PMC5547610

[ref95] VanderWeele T. J. Chen Y. Long K. Kim E. S. Trudel-Fitzgerald C. Kubzansky L. D. (2020). Positive epidemiology? Epidemiology 31, 189–193. doi: 10.1097/EDE.0000000000001147 31809344

[ref96] VanderWeele T. J. McNeely E. Koh H. K. (2019). Reimagining health—flourishing. JAMA 321, 1667–1668. doi: 10.1001/jama.2019.3035, PMID: 30933213

[ref97] VanderWeele T. J. Trudel-Fitzgerald C. Allin P. Farrelly C. Fletcher G. Frederick D. E.. (2020). Current recommendations on the selection of measures for well-being. Prev. Med. 133:106004. doi: 10.1016/j.ypmed.2020.106004 32006530

[ref98] Verme P. (2011). Life satisfaction and income inequality. Rev Income Wealth 57, 111–127. doi: 10.1111/j.1475-4991.2010.00420.x

[ref99] Viswanath K. Bekalu M. Dhawan D. Pinnamaneni R. Lang J. Mcloud R. (2021). Individual and social determinants of COVID-19 vaccine uptake. BMC Public Health 21, 21:818. doi: 10.1186/s12889-021-10862-1, PMID: 33910558 PMC8081000

[ref100] Viswanath K. Emmons K. M. (2006). Message effects and social determinants of health: its application to cancer disparities. J. Commun. 56, S238–S264. doi: 10.1111/j.1460-2466.2006.00292.x

[ref101] Weinstein E. (2018). The social media see-saw: positive and negative influences on adolescents’ affective well-being. New Media Soc. 20, 3597–3623. doi: 10.1177/1461444818755634

[ref102] Willen S. S. (2021). Flourishing and health in critical perspective: an invitation to interdisciplinary dialogue. SSM Ment Health 2:100045. doi: 10.1016/j.ssmmh.2021.100045

[ref103] Witten H. Savahl S. Adams S. Adolescent flourishing: a systematic review. Gugliandolo M. C., editor. Cogent Psychol., (2019). 6:1640341. doi:10.1080/23311908.2019.1640341

[ref104] Xia W. Wai Li L. M. (2022). When and how to share? The role of inspiration. J. Soc. Psychol., 1–15. doi: 10.1080/00224545.2022.2080038 35659508

[ref105] Zhao M. Y. Tay L. (2022). From ill-being to well-being: Bipolar or bivariate? The Journal of Positive Psychology, 1–11. doi: 10.1080/17439760.2022.2109204

