# Supplementary material for: Social media use and adolescents’ well-being: A note on flourishing
Source: Front Psychol. 2023 Apr 6;14:1092109. doi: 10.3389/fpsyg.2023.1092109 (PMC10116992; doi:10.3389/fpsyg.2023.1092109)
Supplement: Supplementary file 1 [file Data_Sheet_1.docx]

***Supplementary material***

| **Table 1.** List of items in the questionnaire and response options. |
| --- |
| **Fluorishing**  ***Fluorishing***  Per favore, rispondi alle seguenti domanda su una scala da 0 a 10:  *Please respond to the following questions on a scale from 0 to 10:*    In generale, quanto sei soddisfatto/a della tua vita nel suo complesso in questi giorni?  *Overall, how satisfied are you with life as a whole these days?*  (0) Per nulla soddisfatto/a (10) Completamente soddisfatto/a  *(0) Not satisfied at All (10) Completely satisfied*  In generale, mi considero una persona felice.  *In general I consider myself a happy person.*  (0) Completamente in disaccordo (10) Completamente d’accordo  *(0) Strongly disagree (10) Strongly agree*  In generale, come valuteresti la tua salute fisica?  *In general, how would you rate your physical health?*  (0) Scarsa (10) Eccellente  *(0) Poor (10) Excellent*  In generale, come valuteresti la tua salute mentale?  *In general, how would you rate your mental health?*  (0) Scarsa (10) Eccellente  *(0) Poor (10) Excellent*  In generale, quanto ritieni che le cose che fai nella tua vita “valgano la pena”?  *Overall, to what extent do you feel the things you do in your life are worthwhile?*  (0) Per nulla (10) Del tutto  *(0) Not at all worthwhile (10) Completely worthwhile*  Le cose che sto facendo ora mi aiuteranno a raggiungere i miei obiettivi di vita.  *I am doing things now that will help me achieve my goals in life.*    (0) Completamente in disaccordo (10) Completamente d’accordo  *(0) Strongly disagree (10) Strongly agree*  Cerco sempre di agire per promuovere il bene in tutte le circostanze, anche in situazioni difficili e impegnative.  *I always act to promote good in all circumstances, even in difficult and challenging situations.*    (0) Assolutamente falso (10) Assolutamente vero  *(0) Not true of me (10) Completely true of me*  Sono sempre stato in grado di rinunciare a un po’ di felicità oggi per una felicità maggiore domani.  *I am always able to give up some happiness now for greater happiness later.*    (0) Assolutamente falso (10) Assolutamente vero  *(0) Not true of me (10) Completely true of me*  Sono soddisfatto/a delle mie amicizie e relazioni.  *I am content with my friendships and relationships.*  (0) Completamente in disaccordo (10) Completamente d’accordo  *(0) Strongly disagree (10) Strongly agree*  Nella mia vita ho delle persone con cui posso parlare delle cose che contano davvero.  *I have people in my life I can talk to about things that really matter.*  (0) Completamente in disaccordo (10) Completamente d’accordo  *(0) Strongly disagree (10) Strongly agree*  La mia famiglia ha abbastanza soldi per vivere una vita dignitosa.  *My family has enough money to live a truly decent life.*  (0) Completamente in disaccordo (10) Completamente d’accordo  *(0) Strongly disagree (10) Strongly agree*  Quanto spesso ti preoccupi della tua sicurezza, del cibo, e dell’alloggio?  *How often do you worry about safety, food, or housing?*  (0) Sempre preoccupato/a (10) Mai stato/a preoccupato/a  *(0) Worry all the time (10) Do not ever worry* |
|  |
| **Esperienze sociali online positive e negative** |
| *Positive and negative online social experiences* |
| Ora indica quanto sei d'accordo con le seguenti affermazioni: "Sui social media..."  *Now indicate how much you agree with the following statements: "On social media ..."* |
|  |
| 1. Completamente in disaccordo (2) Abbastanza in disaccordo (3) Un po’ in disaccordo   (4) Né in disaccordo né in accordo (5) Un po’ in accordo (6) Abbastanza in accordo  (7) Completamente in accordo |
| *(1) Completely disagree (2) Fairly disagree (3) Somewhat disagree*  *(4) Neither disagree nor agree (5) Somewhat agree (6) Fairly agree*  *(7) Completely agree* |
|  |
| Ci sono persone che credono in me e nelle mie abilità |
| *There are people who have faith in me and my abilities.* |
|  |
| Qualcuno mi ha incoraggiato quando sentivo che volevo mollare.  *Someone has encouraged me when I felt like quitting*.  Le persone si complimentano con me per i miei traguardi.  *People praise me for my accomplishments*.  Quando mi sento solo/a, ci sono persone con cui posso parlare.  *When I feel lonely, there are several people I can talk to.*  Appartengo a gruppi di persone con interessi simili ai miei.  *I belong to groups of people with similar interests to mine.*  Le persone considerano poco le mie emozioni.  *People have little regard for my emotions.*  Qualcuno mi ha fatto sentire indesiderato/a.  *Someone has made me feel unwanted.*  Mi sono sentito/a ignorato/a o poco importante per gli altri.  *I felt ignored or unimportant to others.*  Mi sono sentito/a escluso/a dagli altri (es. ho visto degli amici a un evento senza di me).  *I have felt excluded by others. (For example, seeing friends at an event without me.)* |
| **Self-disclosure on social media** |
| ***Self-disclosure on social media*** |
|  |
| Ora indica quanto spesso ti capitano le seguenti situazioni quando esprimi te stesso/a sui social media  *Now indicate how often the following situations happen to you when expressing yourself on social media* |
| Con gli **amici più stretti** (es. migliori amici, coloro con cui interagisci tutti i giorni) |
| *With* ***close friends*** *(e.g. best friends, those you interact with every day)* |
| Con **conoscenti/persone famose** (es. quelle persone che conosci “di vista” oppure blogger, youtuber, influencer) |
| *With* ***acquaintances / famous people*** *(eg. Those people you know "by sight" or bloggers, youtubers, influencers)* |
| Risposte  *Answer options*  (1) Mai (2) A volte (3) Spesso (4) Sempre  *(1) Never (2) Sometimes (3) Often (4) Always* |
| Sono onesto/a quando esprimo qualcosa di me.  *I am honest when I express something about myself.*  Esprimo il mio stato d’animo e i miei sentimenti.  *I express my mood and my feelings*. |
| **Ispirazione sui social media** |
| ***Social media inspiration*** |
|  |
| Sui social media, **se noto che una persona è messa meglio di me**...  *On social media,* ***if I notice that a person is better than me****…*  (0) Mai (10) Sempre  *(0) Never (10) Always* |
| Cerco di migliorarmi.  *I try to ameliorate myself.*  Mi focalizzo su come posso avere successo in futuro allo stesso modo.  *I focus on how I can become equally successful in the future.*  Mi sforzo di raggiungere gli stessi traguardi.  *I strive to reach the same achievements*  Mi sento ispirato/a  *I feel inspired*  *--*  Sui social media, **se noto che io sono messo/a meglio in qualcosa rispetto agli altri**:  *On social media,* ***if I notice that I am better at something than others***…  (0) Mai (10) Sempre  *(0) Never (10) Always*  Cerco di aiutare gli altri a migliorarsi.  *I try to help others improve themselves.*  Mi focalizzo su come posso aiutare gli altri ad avere successo in futuro allo stesso modo.  *I focus on how I can help others be successful in the future in the same way.*  Mi sforzo di aiutare gli altri a raggiungere gli stessi traguardi.  *I strive to help others achieve the same goals*.  Mi sento d'ispirazione per gli altri.  *I feel an inspiration for others.* |
| **Autostima** |
| ***Self-esteem*** |
|  |
| Per favore, indica quanto sei in accordo o disaccordo con ciascuna delle seguenti affermazioni:  *Please indicate how much you agree or disagree with each of the following statements:*  Risposte  *Answer options*  (1) Completamente in disaccordo (2) Abbastanza in disaccordo (3) Abbastanza in accordo  (4) Completamente d’accordo  *(1) Completely disagree (2) Fairly disagree (3) Fairly agree*  *(4) Completely agree*  Nel complesso, sono soddisfatto/a di me stesso/a.  *On the whole, I am satisfied with myself.*  A volte penso di non essere del tutto bravo/a.  *At times I think I am no good at all.*  Sento di avere numerose buone qualità.  *I feel that I have a number of good qualities*.  Sono capace di fare le cose bene come molte altre persone.  *I am able to do things as well as most other people.*  Sento di non avere molto di cui essere orgoglioso/a.  *I feel I do not have much to be proud of.*  A volte mi sento inutile.  *I certainly feel useless at times*.  Sento di essere una persona di valore, se mi confronto con gli altri.  *I feel that I'm a person of worth, at least on an equal plane with others.*  Desidero poter avere più rispetto per me stesso/a.  *I wish I could have more respect for myself*.  Tutto sommato, sento di essere un fallimento.  *All in all, I am inclined to feel that I am a failure.*  Ho un atteggiamento positivo verso me stesso/a.  *I take a positive attitude toward myself*. |
|  |
| **Malessere** |
| ***Ill-being***  Durante l’ultimo mese, quanto spesso ti sono capitate le seguenti cose?  *During the last month, how often have the following things happened to you?*  Risposte  *Answer options*  (1) Mai (2) Raramente (meno di un giorno o due) (3) Per alcuni giorni  (4) Per più della metà dei giorni (5) Quasi ogni giorno  *(1) Never (none) (2) Rarely (slight) (3) Several days (mild)*  *(4) More than half the days (moderate) (5) Almost every day (severe)*  Ho avuto dolori allo stomaco, alla testa o altri dolori o malesseri.  *I have been bothered by stomachaches, headaches, or other aches and pains.*  Ho avuto difficoltà a prestare attenzione mentre ero in classe, facevo i compiti, leggevo o giocavo.  *I have been bothered by not being able to pay attention when I was in class or doing homework or reading a book or playing a game.*  Ho avuto difficoltà ad addormentarmi o a dormire tutta la notte.  *I had a hard time falling asleep or sleeping through the night.*  Mi sono distratto/a facilmente.  *I got distracted easily.*  Mi sono sentito/a in ansia, irrequieto/a, agitato/a.  *I felt nervous, anxious, or scared.*  Non riuscivo a smettere di preoccuparmi per quello che dovevo fare (es. per la scuola).  *I couldn't stop worrying about what I had to do (eg for school).*  Ho provato meno piacere a fare ciò che svolgo di solito.  *I had less fun doing things than you used to.*  Mi sono sentito/a triste o depresso/a per diverse ore.  *I felt sad or depressed for several hours.* |
| **Personalità** |
| ***Personality***  Ti vedi come una persona che...  *I see myself as a person who…*  Risposte  *Answer options*   1. = Mai/*Never* (10) = Sempre/*Always*   ... è riservata?  *… is reserved*  ... si agita facilmente?  *… gets nervous easily*  ... è rilassata, sopporta bene lo stress?  *... is relaxed, handles stress well*  ... è spigliata, socievole?  *… is outgoing, sociable* |
| **Altre variabili di controllo** |
| ***Other control variables***  **Genere**  ***Gender***  Sei...  *You're ...*  (1) Un ragazzo (2) Una ragazza  *(1) A boy (2) A girl*  **Stato socio-economico**  ***Perceived socio-economic status (SES)***  In che misura pensi che la tua famiglia stia bene economicamente?  *How much wealthy do you think your family is?*    (1) Molto bene (2) Bene (3) Mediamente bene  (4) Non tanto bene (5) Per niente bene  *(1) Definitely wealthy (2) Wealthy (3) Average wealthy*  *(4) Not so wealthy (5) Definitely not wealthy*  **Anno scolastico frequentato**  ***High school year attended***  Che anno frequenti?  *Which year do you attend?*  (1) Primo (2) Secondo  *(1) First (2) Second*  **Età**  ***Age***  Quanti anni hai? _____  *How old are you?_____*  **Ambiente scolastico favorevole**  ***Perceived school environment***  In generale, ti trovi bene al tuo liceo (es. con i compagni, insegnanti, attività)?  *In general, do you feel comfortable in your high school (e.g. with peers, teachers, activities)?*  (1) Per nulla (2) Poco (3) Abbastanza (4) Molto  *(1) Not at all (2) Little (3) Enough (4) A lot*  **Attività fisica**  ***Physical activity***  Quanto ti reputi sportivo/a?  *How much do you consider yourself a sporty person?*  (0) Per nulla (10) Moltissimo  *(0) Not at all (10) Very much*  **Stress percepito**  ***Perceived stress***  Durante l'ultimo mese, qual è stato il tuo livello di stress quotidiano?  *During the last month, what was your daily stress level?*  (0) Per nulla stressato/a (10) Molto stressato/a  *(0) Not stressed at all (10) Very stressed* |
|  |

**Table 2. Confirmatory Factor Analysis of the Adolescent Flourishing scale.**

| Flourishing scale | B | SE | β | Z | p-value |
| --- | --- | --- | --- | --- | --- |
| Item_1 | 1 | 1.882 | na | 0.816 | na |
| Item_2 | 1.031 | 0.031 | 0.847 | 33.789 | <.001 |
| Item_3 | 0.576 | 0.034 | 0.526 | 16.84 | <.001 |
| Item_4 | 1.103 | 0.036 | 0.788 | 30.348 | <.001 |
| Item_5 | 0.833 | 0.035 | 0.733 | 23.79 | <.001 |
| Item_6 | 0.638 | 0.035 | 0.580 | 18.189 | <.001 |
| Item_7 | 0.480 | 0.034 | 0.460 | 14.138 | <.001 |
| Item_8 | 0.382 | 0.037 | 0.322 | 10.271 | <.001 |
| Item_9 | 0.593 | 0.034 | 0.518 | 17.18 | <.001 |
| Item_10 | 0.558 | 0.041 | 0.458 | 13.771 | <.001 |
| Item_11 | 0.295 | 0.029 | 0.364 | 10.256 | <.001 |
| Item_12 | 0.421 | 0.049 | 0.249 | 8.513 | <.001 |

Legend. B = estimate; SE = standard errors; Z = z-value; *β* = standardized coefficient; na = not applicable. Estimator used= maximum likelihood estimation with robust standard errors and a Satorra-Bentler scaled test statistic. (χ^2^= 398.387, df=53, p <.001, CFI=.914, RMSEA=. 068, SRMR=.046). Item 9 and 10 were allowed to correlate.

**Table 3. Additional regression analysis with Flourishing as the outcome.**

|  | **Model 1** | | | |
| --- | --- | --- | --- | --- |
|  | **B** | **(SE)** | **Beta** | **p** |
| Constant | 6.544 | .195 |  | .000 |
| *Positive online experiences* |  |  |  |  |
| There are people who have faith in me and my abilities. | .108 | .029 | .118 | .000 |
| Someone has encouraged me when I felt like quitting. | -.067 | .024 | -.083 | .006 |
| People praise me for my accomplishments. | .102 | .025 | .114 | .000 |
| When I feel lonely, there are several people I can talk to. | .120 | .025 | .146 | .000 |
| I belong to groups of people with similar interests to mine. | .013 | .024 | .014 | .593 |
| *Negative online experiences* |  |  |  |  |
| People have little regard for my emotions. | -.058 | .025 | -.064 | .000 |
| Someone has made me feel unwanted. | -.168 | .023 | -.211 | .019 |
| I felt ignored or unimportant to others. | -.101 | .027 | -.124 | .000 |
| I have felt excluded by others. (For example, seeing friends at an event without me.) | -.044 | .024 | -.056 | .000 |
| *Inspiration from others* |  |  |  |  |
| I try to ameliorate myself. | .037 | .017 | .068 | .029 |
| I focus on how I can become equally successful in the future. | -.032 | .019 | -.060 | .091 |
| I strive to reach the same achievements | .029 | .018 | .055 | .106 |
| I feel inspired | .062 | .016 | .111 | .000 |
| Adjusted R square | .286** | | | |

Legend : B=Unstandardized beta, (SE)= standard error, β=Standardized beta, p=p-value
